# Supplementary material for: Sectoral linkages and their influence on structural change: The case of China
Source: PLoS One. 2025 Sep 3;20(9):e0330908. doi: 10.1371/journal.pone.0330908 (PMC12407444; doi:10.1371/journal.pone.0330908)
Supplement: S1 Appendix — (DOCX) [file pone.0330908.s001.docx]

**Appendix**

In the appendix, we provide a detailed explanation of how to solve the general equilibrium problem defined in our model to derive the expression for sectoral value-added shares. In Part 2, we outline the process of obtaining the equation that defines the relationship between the derivation of sectoral productivities and sectoral value-added. Furthermore, we explore how sectoral linkages influence the dynamics of value-added from a network perspective.

## A. Sectoral linkages and value-added share

In a competitive equilibrium, the following conditions hold: (1) households maximize their utility while taking prices of goods as given; (2) firms maximize their profits while taking factor prices as given; and (3) all goods and factor markets are cleared.

By solving the household's optimization problem, we obtain:

$P=\left( \omega_{a}P_{a}^{1-\varepsilon}+\omega_{m}P_{m}^{1-\varepsilon}+\omega_{s}P_{s}^{1-\varepsilon} \right)^{\frac{1}{1-\varepsilon}}$ (1)

$P_{a}\tilde{C_{a}}=\omega_{a}\left( \frac{P_{a}}{P} \right)^{1-\varepsilon}(WL+P_{a}\bar{C_{a}}+P_{s}\bar{C_{s}})$ (2)

$P_{m}\tilde{C_{m}}=\omega_{m}\left( \frac{P_{m}}{P} \right)^{1-\varepsilon}(WL+P_{a}\bar{C_{a}}+P_{s}\bar{C_{s}})$ (3)

$P_{s}\tilde{C_{s}}=\omega_{s}\left( \frac{P_{s}}{P} \right)^{1-\varepsilon}(WL+P_{a}\bar{C_{a}}+P_{s}\bar{C_{s}})$ (4)

P is defined as the price of aggregate consumption C. $\tilde{C_{i}}=C_{i}+\bar{C_{i}}$ for i=a, m, s, and $\bar{C_{m}}=0$.

By solving the firms’ optimization problems, we obtain:

$WL_{i}=\alpha_{i}P_{i}Y_{i}$ (5)

${P_{j}M_{ji}=(1-\alpha}_{i})\mu_{ji}P_{i}Y_{i}$ (6)

So, the dual equation for (6) is:

${P_{i}M_{ij}=(1-\alpha}_{j})\mu_{ij}P_{j}Y_{j}$ (7)

$M_{ij}$ is defined as the domestic intermediate input from sector j used by sector i.

The markets’ clearing condition satisfies:

$Y_{i}=\tilde{C_{i}}+\sum_{j} M_{ij}$ (8)

$\sum_{i} L_{i}=L$ (9)

By multiplying the current price of goods $P_{i}$ in equation (8), we obtain:

$P_{i}Y_{i}=P_{i}\tilde{C_{i}}+P_{i}\sum_{j} M_{ij}$ (10)

By substituting $M_{ij}$ with equation (7) we obtain:

$P_{i}Y_{i}=P_{i}\tilde{C_{i}}+\sum_{j} \mu_{ij}(1-\alpha_{j})P_{j}Y_{j}$ (11)

By substituting the nominal output $P_{i}Y_{i}$and $P_{j}Y_{j}$ with equation (5) we obtain:

$\frac{WL_{i}}{\alpha_{i}}=P_{i}\tilde{C_{i}}+\sum_{j} \mu_{ij}(1-\alpha_{j})\frac{WL_{j}}{\alpha_{j}}$ (12)

By simplifying equation (12) we obtain:

${WL}_{i}={\alpha_{i}P}_{i}\tilde{C_{i}}+\sum_{j} \mu_{ij}(1-\alpha_{j})\frac{\alpha_{i}}{\alpha_{j}}WL_{j}$ (13)

Since the term ${WL}_{i}$ and ${WL}_{j}$ represent the value-added of sector *i* and *j* by the definition of exponential $\alpha_{i}$ and $\alpha_{j}$, so we can denote them as $V_{i}$ and $V_{j}$. Consequently, we have *WL=W* ($\sum_{i} L_{i})=\sum_{i} V_{i}=V$. By combining all three sectors, we can express equation (13) in matrix form as follows:

$\left( \begin{aligned} V_{a} \\ V_{m} \\ V_{s} \end{aligned} \right)=\left[ \begin{matrix} \gamma_{aa} & \gamma_{am} & \gamma_{as} \\ \gamma_{ma} & \gamma_{mm} & \gamma_{ms} \\ \gamma_{sa} & \gamma_{sm} & \gamma_{ss} \end{matrix} \right]\left( \begin{aligned} V_{a} \\ V_{m} \\ V_{s} \end{aligned} \right)+\left( \begin{matrix} \alpha_{a} & 0 & 0 \\ 0 & \alpha_{m} & 0 \\ 0 & 0 & \alpha_{s} \end{matrix} \right)\left( \begin{aligned} P_{a}\tilde{C_{a}} \\ P_{m}\tilde{C_{m}} \\ P_{s}\tilde{C_{s}} \end{aligned} \right)$ (14)

In equation (14), $\gamma_{ij}=\mu_{ij}(1-\alpha_{j})(\frac{\alpha_{i}}{\alpha_{j}})$. The equation (14) can be simplified to:

$\left( \begin{aligned} V_{a} \\ V_{m} \\ V_{s} \end{aligned} \right)=\left[ \begin{matrix} {1-\gamma}_{aa} & {-\gamma}_{am} & {-\gamma}_{as} \\ {-\gamma}_{ma} & 1-\gamma_{mm} & {-\gamma}_{ms} \\ {-\gamma}_{sa} & {-\gamma}_{sm} & 1-\gamma_{ss} \end{matrix} \right]^{-1}\left( \begin{matrix} \alpha_{a} & 0 & 0 \\ 0 & \alpha_{m} & 0 \\ 0 & 0 & \alpha_{s} \end{matrix} \right)\left( \begin{aligned} P_{a}\tilde{C_{a}} \\ P_{m}\tilde{C_{m}} \\ P_{s}\tilde{C_{s}} \end{aligned} \right)$

$=\left[ \begin{matrix} \Omega_{aa} & \Omega_{am} & \Omega_{as} \\ \Omega_{ma} & \Omega_{mm} & \Omega_{ms} \\ \Omega_{sa} & \Omega_{sm} & \Omega_{ss} \end{matrix} \right]\left( \begin{aligned} P_{a}\tilde{C_{a}} \\ P_{m}\tilde{C_{m}} \\ P_{s}\tilde{C_{s}} \end{aligned} \right)$ (15)

Plug the expressions for final demand from equations (2) to (4) into the equation and simplify. We then obtain:

$\frac{V_{i}}{V}=\left( 1+\frac{P_{a}\bar{C_{a}}}{V}+\frac{P_{s}\bar{C_{s}}}{V} \right)[\sum_{j} \Omega_{ij}\omega_{j}\left( \frac{P_{j}}{P} \right)^{1-\varepsilon}]-\Omega_{ia}\frac{P_{a}\bar{C_{a}}}{V}-\Omega_{is}\frac{P_{s}\bar{C_{s}}}{V}$, for i=a, m, s. (16)

The equation (16) provides the expression for value-added shares in general equilibrium, along with its relationship to sectoral linkages and prices.

## B. Nonlinear least square method in calibration

We use nonlinear least square (NLS) method to calibrate parameters. Taking the manufacturing sector as the reference, the objective function for calibration is:

${min}_{\left\{ \omega_{i},\varepsilon\right\}}\sum_{t} \sum_{i\in\left\{ a, s \right\}} \left[ \left( \frac{P_{it}C_{it}}{P_{mt}} \right)-\left( \frac{E_{it}}{E_{mt}} \right) \right]^{2}$ (17)

The final absorption percentage among sectors derived from first-order conditions of household’s optimization problem (equation 2-4) implies:

$\frac{P_{i}\tilde{C_{i}}}{P_{m}\tilde{C_{m}}}=\frac{\omega_{i}}{\omega_{m}}\left( \frac{P_{i}}{P_{m}} \right)^{1-\varepsilon}$, for i=a, s. (18)

Since $P_{i}\tilde{C_{i}}=P_{i}C_{i}+P_{i}\bar{C_{i}}$, $\bar{C_{m}}=0$, it can be simplified as:

$\frac{P_{i}C_{i}}{P_{m}C_{m}}=\frac{\omega_{i}}{\omega_{m}}\left( \frac{P_{i}}{P_{m}} \right)^{1-\varepsilon}-\frac{P_{i}\bar{C_{i}}}{P_{m}C_{m}}$ (19)

Equation (3) implies that:

$P_{m}\tilde{C_{m}}=P_{m}C_{m}=\omega_{m}\left( \frac{P_{m}}{P} \right)^{1-\varepsilon}\left( WL+P_{a}\bar{C_{a}}+P_{s}\bar{C_{s}} \right)$

$=\omega_{m}\left( \frac{P_{m}}{P} \right)^{1-\varepsilon}\left( V+P_{a}\bar{C_{a}}+P_{s}\bar{C_{s}} \right)$ (20)

Take equation (20) into equation (19) and then simplify it we obtain:

$\frac{P_{i}C_{i}}{P_{m}C_{m}}=\frac{\omega_{i}}{\omega_{m}}\left( \frac{P_{i}}{P_{m}} \right)^{1-\varepsilon}-\frac{P_{i}\bar{C_{i}}}{\omega_{m}\left( V+P_{a}\bar{C_{a}}+P_{s}\bar{C_{s}} \right)}\left( \frac{P}{P_{m}} \right)^{1-\varepsilon}$ (21)

Take equation (21) into equation (17), the final calibration equation is illustrated as follows:

${min}_{\{\omega_{i},\varepsilon\}}\sum_{t} \sum_{i\in\{a, s\}} {[\left( \frac{\omega_{i}}{\omega_{m}} \right)\left( \frac{P_{it}}{P_{mt}} \right)^{1-\varepsilon}-\frac{P_{it}\bar{C_{i}}}{\omega_{m}\left( V+P_{at}\bar{C_{a}}+P_{st}\bar{C_{s}} \right)}\left( \frac{P_{t}}{P_{mt}} \right)^{1-\varepsilon}-(\frac{E_{it}}{E_{mt}})]}^{2}$ (22)

## C. Network effects of sectoral linkages

In the supply side, we take the logarithm and then perform a total differentiation of equations (8) and (9). This yields equations (23) and (24) presented below.

$dlnW+dlnL_{i}=dlnP_{i}+dlnY_{i}$ (23)

$dlnP_{j}+dlnM_{ji}=dlnP_{i}+dlnY_{i}$ (24)

In the demand side, we apply the same methodology as described above to derive equation (25).

$dlnP_{i}=\frac{1}{\varepsilon}dlnW-\frac{1-\varepsilon}{\varepsilon}dlnP-\frac{1}{\varepsilon}dln\tilde{C_{i}}$ (25)

For the production function, we utilize the same approach previously mentioned, which enables us to obtain equation (26).

$dlnY_{i}=dZ_{i}+\alpha_{i}dlnL_{i}+(1-\alpha_{i})\sum_{j} \mu_{ji}dlnM_{ji}$ (26)

Plug equations (23) through (25) into equation (26), then we obtain:

$\left( \begin{aligned} dln\tilde{C_{a}} \\ dln\tilde{C_{m}} \\ dln\tilde{C_{s}} \end{aligned} \right)=\varepsilon\left( \begin{aligned} {dZ}_{a} \\ {dZ}_{m} \\ {dZ}_{s} \end{aligned} \right)$ $+\left( 1-\varepsilon\right)\left[ \begin{matrix} \alpha_{a} & 0 & 0 \\ 0 & \alpha_{m} & 0 \\ 0 & 0 & \alpha_{s} \end{matrix} \right]\left( dlnW-dlnP \right)$

$+\left[ \begin{matrix} \mu_{aa}\left( 1-\alpha_{a} \right) & \mu_{ma}\left( 1-\alpha_{a} \right) & \mu_{sa}\left( 1-\alpha_{a} \right) \\ \mu_{am}\left( 1-\alpha_{m} \right) & \mu_{mm}\left( 1-\alpha_{m} \right) & \mu_{sm}\left( 1-\alpha_{m} \right) \\ \mu_{as}\left( 1-\alpha_{s} \right) & \mu_{ms}\left( 1-\alpha_{s} \right) & \mu_{ss}\left( 1-\alpha_{s} \right) \end{matrix} \right]\left( \begin{aligned} dln\tilde{C_{a}} \\ dln\tilde{C_{m}} \\ dln\tilde{C_{s}} \end{aligned} \right)$,

Simplify the equation above and express it in vector form:

$\boldsymbol{dln}\tilde{\boldsymbol{C}}\boldsymbol{=}\varepsilon\boldsymbol{\Omega}_{\boldsymbol{\mu}}\boldsymbol{dZ+}(1-\varepsilon)\boldsymbol{\Lambda}_{\boldsymbol{\alpha}}\boldsymbol{(dlnW-dlnP)}$. While $\boldsymbol{dln}\tilde{\boldsymbol{C}}\boldsymbol{=}\left( \begin{aligned} dln\tilde{C_{a}} \\ dln\tilde{C_{m}} \\ dln\tilde{C_{s}} \end{aligned} \right)$,

$\boldsymbol{dZ}=\left( \begin{aligned} {dZ}_{a} \\ {dZ}_{m} \\ {dZ}_{s} \end{aligned} \right)$,$\boldsymbol{\Lambda}_{\boldsymbol{\alpha}}=\left( \begin{matrix} \alpha_{a} & 0 & 0 \\ 0 & \alpha_{m} & 0 \\ 0 & 0 & \alpha_{s} \end{matrix} \right)$,

$\boldsymbol{\Omega}_{\boldsymbol{\mu}}=\left[ \begin{matrix} {1-\mu}_{aa}\left( 1-\alpha_{a} \right) & {-\mu}_{ma}\left( 1-\alpha_{a} \right) & -\mu_{sa}\left( 1-\alpha_{a} \right) \\ -\mu_{am}\left( 1-\alpha_{m} \right) & {1-\mu}_{mm}\left( 1-\alpha_{m} \right) & -\mu_{sm}\left( 1-\alpha_{m} \right) \\ -\mu_{as}\left( 1-\alpha_{s} \right) & {-\mu}_{ms}\left( 1-\alpha_{s} \right) & 1-\mu_{ss}\left( 1-\alpha_{s} \right) \end{matrix} \right]^{-1}$.

Vector $\boldsymbol{dlnW-dlnP}$ has identical entries, specifically denoted as $dlnW-dlnP$.

By applying the same method above for equation (11) we obtain:

$\boldsymbol{dlnY}\approx\boldsymbol{\Lambda}_{\boldsymbol{e}}\boldsymbol{dln}\tilde{\boldsymbol{C}}$ **=** $\boldsymbol{\Gamma}_{\boldsymbol{z}}\boldsymbol{dZ+}\boldsymbol{\Gamma}_{\boldsymbol{p}}\left( \boldsymbol{dlnW-dlnP} \right)$. While $\boldsymbol{\Lambda}_{\boldsymbol{e}}=\left( \begin{matrix} \frac{2-\varepsilon}{\varepsilon} & \frac{\varepsilon-1}{\varepsilon} & \frac{\varepsilon-1}{\varepsilon} \\ \frac{\varepsilon-1}{\varepsilon} & \frac{2-\varepsilon}{\varepsilon} & \frac{\varepsilon-1}{\varepsilon} \\ \frac{\varepsilon-1}{\varepsilon} & \frac{\varepsilon-1}{\varepsilon} & \frac{2-\varepsilon}{\varepsilon} \end{matrix} \right)$, $\boldsymbol{\Gamma}_{\boldsymbol{z}}=\varepsilon\boldsymbol{\Lambda}_{\boldsymbol{e}}\boldsymbol{\Omega}_{\boldsymbol{\mu}}$, $\boldsymbol{\Gamma}_{\boldsymbol{p}}=(1-\varepsilon\boldsymbol{)\Lambda}_{\boldsymbol{e}}\boldsymbol{\Lambda}_{\boldsymbol{\alpha}}$**.**

Since $Y_{i}=\alpha_{i}V_{i}$, so we have $\boldsymbol{dlnY=dlnV}$.
